# Supplementary material for: Screen-Printed Flexible Thermoelectric Device Based on Hybrid Silver Selenide/PVP Composite Films
Source: Nanomaterials (Basel). 2021 Aug 11;11(8):2042. doi: 10.3390/nano11082042 (PMC8401139; doi:10.3390/nano11082042)
Supplement: Supplementary file 1 [file nanomaterials-11-02042-s001.zip › nanomaterials-1321391-supplementary.pdf]

Supplementary Material

Screen-printed Flexible Thermoelectric Device Based on Hybrid Silver Selenide/PVP Composite Films

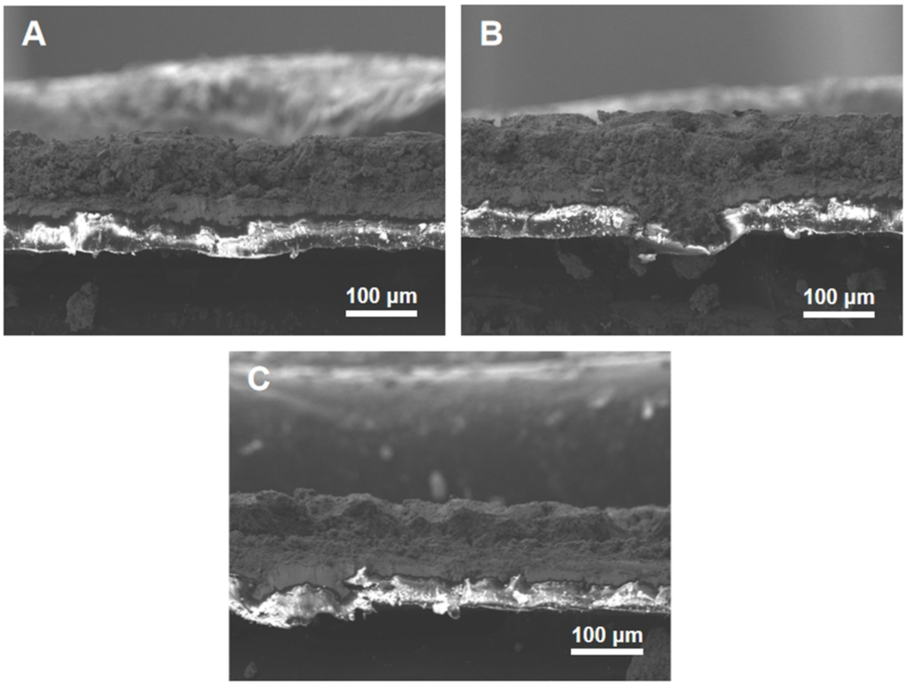

Figure S1. The field emission scanning electron microscope of PI10, PI20 and PI30 film thicknesses

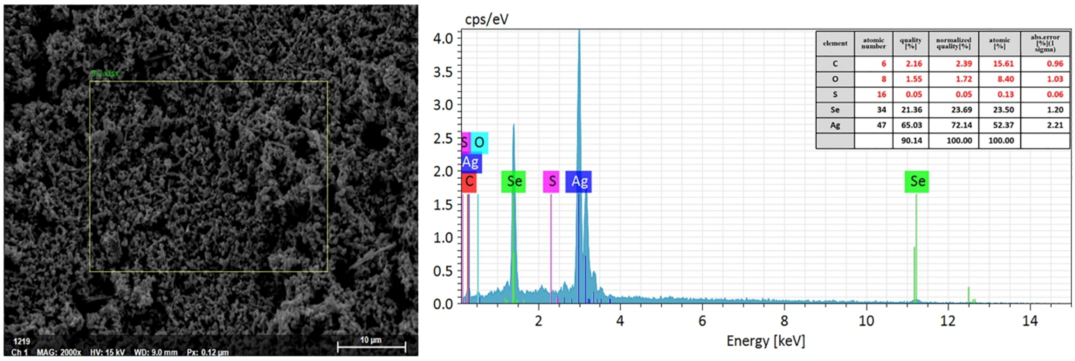

element	atomic number	quality [%]	normalized quality[%]	atomic [%]	abs.error [%](1 sigma)
C	6	2.16	2.39	15.61	0.96
O	8	1.55	1.72	8.40	1.03
S	16	0.05	0.05	0.13	0.06
Se	34	21.36	23.69	23.50	1.20
Ag	47	65.03	72.14	52.37	2.21
		90.14	100.00	100.00	

Figure S2. The energy dispersive spectrum analysis of the PI30 film

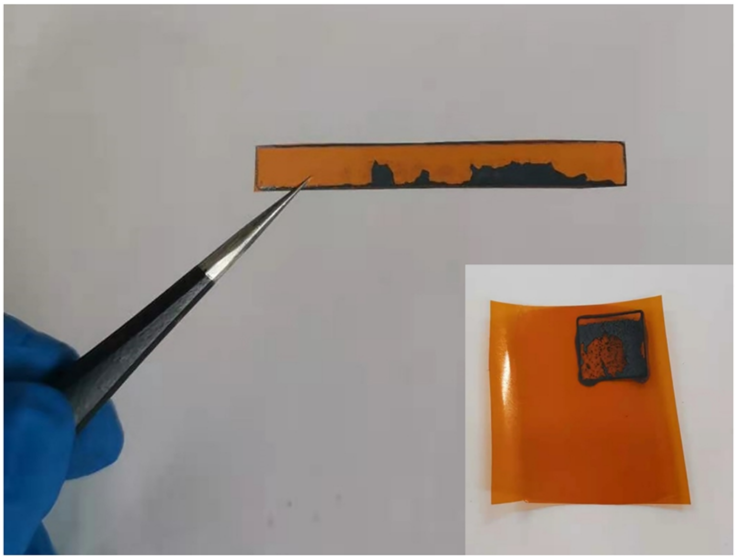

Figure S3. The image of a dry TEG module no longer adhering to the PI substrate

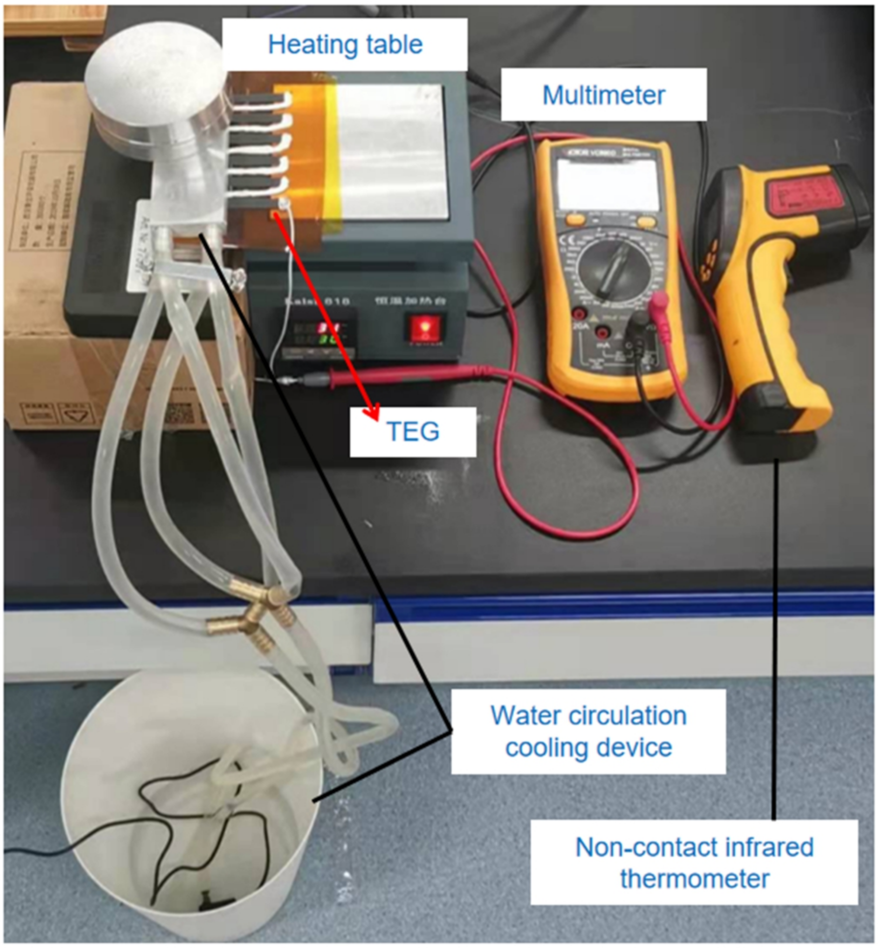

Figure S4. The image of F-TEG output performance measurement circuit
